# Dietary Docosahexaenoic Acid and Eicosapentaenoic Acid Influence Liver Triacylglycerol and Insulin Resistance in Rats Fed a High-Fructose Diet

**DOI:** 10.3390/md13041864

**Published:** 2015-04-01

**Authors:** Gabriela Salim de Castro, Rafael Deminice, Livia Maria Cordeiro Simões-Ambrosio, Philip C. Calder, Alceu A. Jordão, Helio Vannucchi

**Affiliations:** 1 Department of Internal Medicine, Faculty of Medicine of Ribeirão Preto, University of São Paulo, Av. Bandeirantes 3900, Ribeirão Preto SP 14 049-900, Brazil; E-Mails: livia@fmrp.usp.br (L.M.C.S.-A.); alceu@fmrp.usp.br (A.A.J.); hvannucc@fmrp.usp.br (H.V.); 2 Human Development and Health Academic Unit, Faculty of Medicine, University of Southampton, Southampton SO16 6YD, UK; E-Mail: pcc@soton.ac.uk; 3 Department of Physical Education, Faculty of Physical Education and Sport, State University of Londrina, Rodovia Celso Garcia Cid, Pr 445 Km 380, Campus Universitário, Londrina, Paraná 86057-970, Brazil; E-Mail: rdeminice@uel.br

**Keywords:** fish oil, fructose, metabolic syndrome, omega-3 fatty acids

## Abstract

This study aimed to examine the benefits of different amounts of omega-3 (*n*-3) polyunsaturated fatty acids from fish oil (FO) on lipid metabolism, insulin resistance and gene expression in rats fed a high-fructose diet. Male Wistar rats were separated into two groups: Control (C, *n* = 6) and Fructose (Fr, *n* = 32), the latter receiving a diet containing 63% by weight fructose for 60 days. After this period, 24 animals from Fr group were allocated to three groups: FrFO2 (*n* = 8) receiving 63% fructose and 2% FO plus 5% soybean oil; FrFO5 (*n* = 8) receiving 63% fructose and 5% FO plus 2% soybean oil; and FrFO7 (*n* = 8) receiving 63% fructose and 7% FO. Animals were fed these diets for 30 days. Fructose led to an increase in liver weight, hepatic and serum triacylglycerol, serum alanine aminotransferase and HOMA1-IR index. These alterations were reversed by 5% and 7% FO. FO had a dose-dependent effect on expression of genes related to hepatic β-oxidation (increased) and hepatic lipogenesis (decreased). The group receiving the highest FO amount had increased markers of oxidative stress. It is concluded that *n*-3 fatty acids may be able to reverse the adverse metabolic effects induced by a high fructose diet.

## 1. Introduction

The omega-6/omega-3 (*n*-6/*n*-3) fatty acid ratio of the human diet is believed to have changed greatly over time. Before industrialization, the estimated *n*-6/*n*-3 ratio was 1:1 and nowadays estimates indicate a typical ratio of 15:1 in Western countries [1]. The ideal ratio is said to be between 2:1 and 4:1 [2,3]. One way to lower the ratio is to consume more *n*-3 fatty acids. At sufficiently high intakes, the long chain *n*-3 fatty acids eicosapentaenoic acid (EPA) and docosahexaenoic acid (DHA) can lower plasma triacylglycerol (TAG) concentrations, reduce cardiovascular morbidity and mortality, and improve rheumatoid arthritis, asthma, and other inflammatory conditions [1,4,5]. Long chain *n*-3 fatty acids incorporated into cell membrane phospholipids can alter cell signaling, insulin sensitivity and gene expression [3]. In the liver, *n*-3 fatty acids stimulate β-oxidation and decrease lipogenesis [6]. In the adipose tissue, they increase fatty acid mobilization and reduce fat storage through peroxisome proliferator activated receptor (PPAR)-γ activation [7]. Lowering the *n*-6/*n*-3 ratio by increasing intake of another *n*-3 fatty acid, α-linolenic acid, does not produce the same response as seen with EPA and DHA [8]. Increased intake of α-linolenic acid generates a limited increase in EPA and no increase in DHA [8], suggesting that the limitation in α-linolenic acid effectiveness is due to poor conversion to its more bioactive derivatives. Lowering the *n*-6/*n*-3 fatty acid ratio by decreasing intake of the main dietary *n*-6 fatty acid, linoleic acid, favors synthesis of EPA from α-linolenic acid, although again this is limited [8]. These observations suggest that the biggest effect from an altered ratio of *n*-6 to *n*-3 fatty acids is seen if the intake of EPA and DHA is increased.

In recent decades the incidence of obesity and its related comorbidities, such as hypertension, dyslipidemia, insulin resistance (IR), diabetes mellitus and non-alcoholic fatty liver disease has increased and gained considerable attention in public health policy and in research [9,10]. The increased consumption of simple carbohydrates, in particular fructose, has gained attention for its close relationship with the aforementioned comorbidities [11].

In laboratory animals and non-human primates high fructose consumption promotes features of metabolic syndrome, such as obesity, hypertriglyceridemia and IR [12,13]. Long chain *n*-3 fatty acids may be able to reverse these alterations, although high amounts of these fatty acids may adversely affect oxidative stress [6,14]. The precise relationship between increasing amount of *n*-3 fatty acids, especially EPA and DHA, in the diet and these various outcomes is not clear. Therefore, the current study investigated the ability of diets with different amounts of EPA and DHA, and consequently different *n*-6/*n*-3 fatty acid ratios, to reverse the metabolic consequences of a high fructose diet in Wistar rats.

## 2. Results

### 2.1. Growth, Body and Tissue Weight and Metabolic Parameters

**Figure 1:**
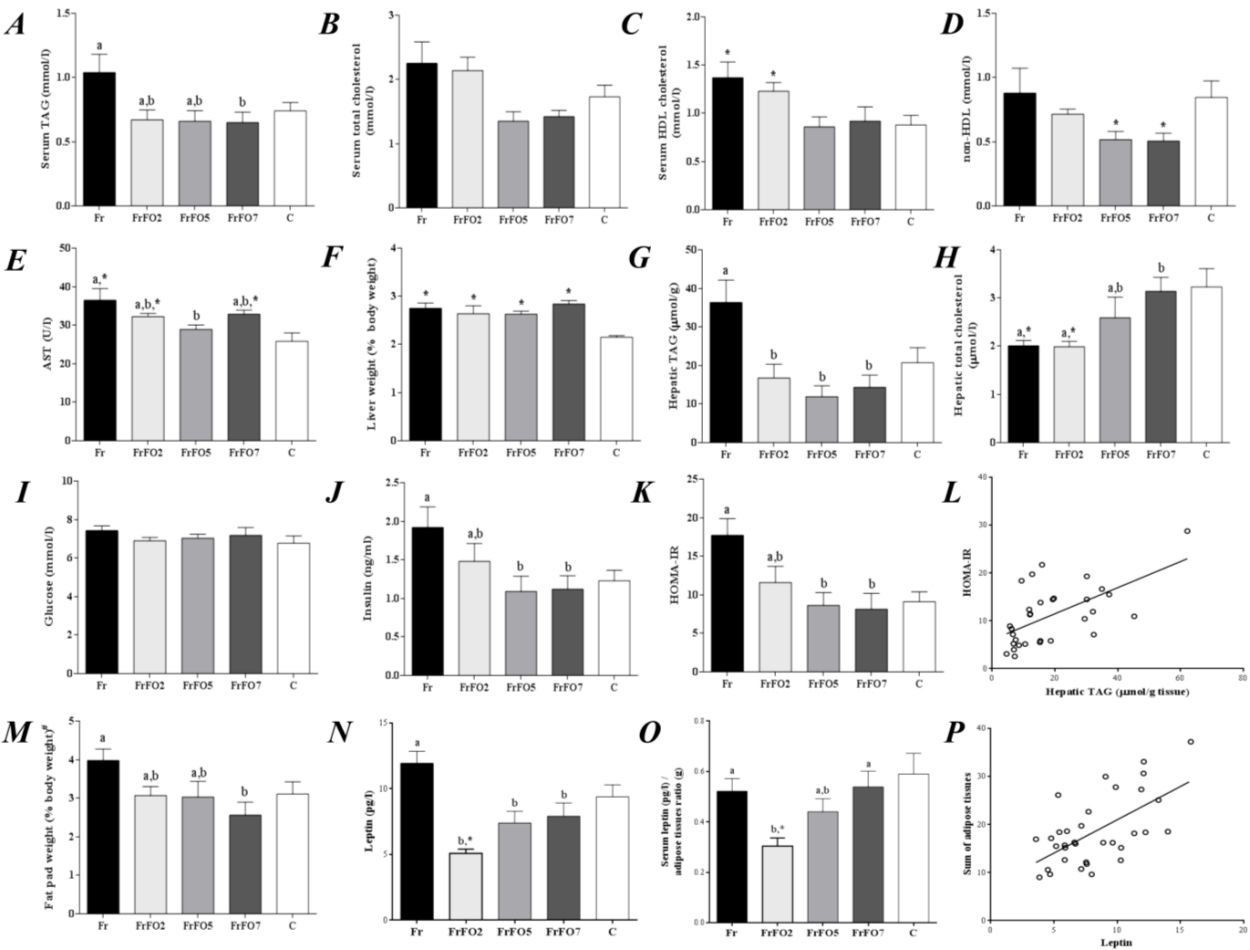
Serum and hepatic parameters after 90 days. (**A**) serum triacylglycerol (TAG); (**B**) serum total cholesterol; (**C**) serum high-density lipoprotein (HDL) cholesterol; (**D**) non-HDL cholesterol; (**E**) serum aspartate aminotransferase (AST); (**F**) liver as a percentage of body weight; (**G**) hepatic TAG; (**H**) hepatic total cholesterol; (**I**) serum glucose; (**J**) serum insulin; (**K**) Homeostatic model assessment (HOMA-IR); (**L**) positive association of HOMA-IR and hepatic TAG (*r* = 0.60, *p* < 0.001); (**M**) adipose tissues (^#^ epididymal and retroperitoneal adipose tissues) as a percentage of body weight; (**N**) serum leptin; (**O**) serum leptin/adipose tissue ratio; (**P**) positive association of serum leptin and sum of adipose tissues (*r* = 0.61, *p* < 0.001). Data are mean ± SEM. Data for the four groups receiving fructose were compared by ANOVA with Tukey’s test; ^a,b^ values not sharing a superscript letter are significantly different (*p* < 0.05). Data for the control group were compared to each group receiving fructose by unpaired t test; * indicates that the fructose group is different from control (*p* < 0.05). Fr—fructose group; FrFO2—fructose and 2% fish oil group; FrFO5—fructose and 5% fish oil group; FrFO7—fructose and 7% fish oil group; C—control group.

Growth and body weight gain did not differ among the dietary groups over the 90 days of the experiment (Table 1). Fr had lower food intake compared to FrFO5 and C; however fructose intake did not differ among the four groups that received the high fructose diet. Fructose consumption increased liver weight in relation to body weight, with no effect of fish oil (Figure 1). Seven percent fish oil decreased fat pad weight (as % body weight) and serum TAG compared to Fr (Figure 1). High density lipoprotein (HDL) cholesterol was higher in Fr and FrFO2 compared to C (Figure 1).

**Table 1:** Weight gain, food consumption and oxidative stress parameters in rats fed different diets.

Parameter	Fr	FrFO2	FrFO5	FrFO7	C
Final body weight (g)	614 ± 30	594 ± 24	588 ± 14	609 ± 22	558 ± 41
Body weight gain (g)	375 ± 30	346 ± 20	333 ± 13	350 ± 23	331 ± 34
Food intake (g/day)	26 ± 1 ^a,^*	28 ± 1 ^a,b^	29 ± 1 ^b^	28 ± 1 ^a,b^	31 ± 2
Fructose intake (g/day) ^#^	16.25 ± 1.18 *	17.35 ± 0.82 *	18.45 ± 0.64 *	17.83 ± 0.88 *	1.56 ± 0.09
Fish oil intake (g/day) ^##^	-	0.59 ± 0.03 ^a^	1.59 ± 0.02 ^b^	2.26 ± 0.02 ^c^	-
EPA+DHA (mg/day)	7 ± 0.1 ^a^	128 ± 5 ^b,^*	328 ± 11 ^c,^*	440 ± 13 ^d,^*	8 ± 0.16
EPA+DHA (mg/day/kg body weight)	15 ± 2 ^a,^*	243 ± 28 ^b,^*	654 ± 56 ^c,^*	917 ± 61 ^d,^*	18 ± 1
**Serum Oxidative Stress Parameters**					
FRAP (μmol/L)	384.5 ± 19.2 ^a^	331.4 ± 11.1 ^a,b,^*	339.5 ± 6.86 ^a,b,^*	317.5 ± 20.6 ^b,^*	390.9 ± 20.24
Hydroperoxides (μmol/L)	40.3 ± 4.1 *	51.8 ± 3.6 *	50.1 ± 2.3 *	53.5 ± 6.5 *	30.6 ± 2.73
TBARS (nmol/g protein)	53.1 ± 4.6	75.7 ± 7.7	69.3 ± 4.0	65.8 ± 5.8	61.0 ± 7.06
GSH (nmol/g protein)	116.9 ± 8.4	116.8 ± 4.8	120.6 ± 6.0 *	125.8 ± 7.8 *	92.8 ± 8.53
**Liver Oxidative Stress Parameters**					
GSH-Red (mU/mg protein)	10.0 ± 3.5	8.1 ± 1.3	8.6 ± 1.0	13.9 ± 1.6	11.2 ± 2.78
GSH-Px (mU/mg protein)	111.8 ± 8.7	102.2 ± 17.0	70.5 ± 7.7 *	106.6 ± 12.7	134.5 ± 27.1
TBARS (nmol/g protein)	409.3 ± 68.8 ª^,b^	198.8 ± 29.1 ^a,^*	311.7 ± 48.1 ^a^	563.8 ± 65.6 ^b^	448.6 ± 101.8
GSH (mmol/g protein)	21.1 ± 1.8	16.4 ± 2.8	15.4 ± 1.6	22.5 ± 1.7	22.2 ± 4.2

^#^ During 90 days; ^##^ last 30 days. Data are mean ± standard error of the mean (SEM.). Data for the four groups receiving fructose were compared by ANOVA with Tukey’s test; ^a,b,c,d^ values across a row not sharing a superscript letter are significantly different (*p* < 0.05). Data for the control group were compared to each group receiving fructose by unpaired *t* test; * indicates that the fructose group is different from control (*p* < 0.05). FRAP—ferric reducing antioxidant power; TBARS—thiobarbituric acid reactive species; GSH—reduced glutathione; GHS-Red—glutathione reductase enzyme activity; GSH-Px—glutathione peroxidase enzyme activity. Fr—fructose group; FrFO2—fructose and 2% fish oil group; FrFO5—fructose and 5% fish oil group; FrFO7—fructose and 7% fish oil group; C—control group.

Fr had higher hepatic TAG than C and inclusion of fish oil in the diet prevented this effect of a high fructose diet (Figure 1). On the other hand, FrFO5 and FrFO7 had higher liver total cholesterol compared to Fr and FrFO2 (Figure 1).

Although fasting glucose was not different among the different groups, Fr had IR, as shown by a higher insulin concentration and the HOMA1-IR (Figure 1). IR was partly reversed by inclusion of fish oil in the diet. Hepatic TAG was positively associated with HOMA-IR (*r* = 0.60, *p* < 0.001; Figure 1). Serum leptin levels were lower in groups that received fish oil compared to Fr. Also, Fr had higher serum AST compared to FrFO5 and C. Serum leptin was positively associated with the sum of adipose tissues (*r* = 0.61, *p* < 0.001; Figure 1).

### 2.2. Oxidative Stress Markers

FrFO7 had lower serum ferric reducing antioxidant power (FRAP) compared to Fr and C and higher liver lipid peroxidation compared to FrFO5 and C (Table 1).

### 2.3. Hepatic Gene Expression

**Figure 2:**
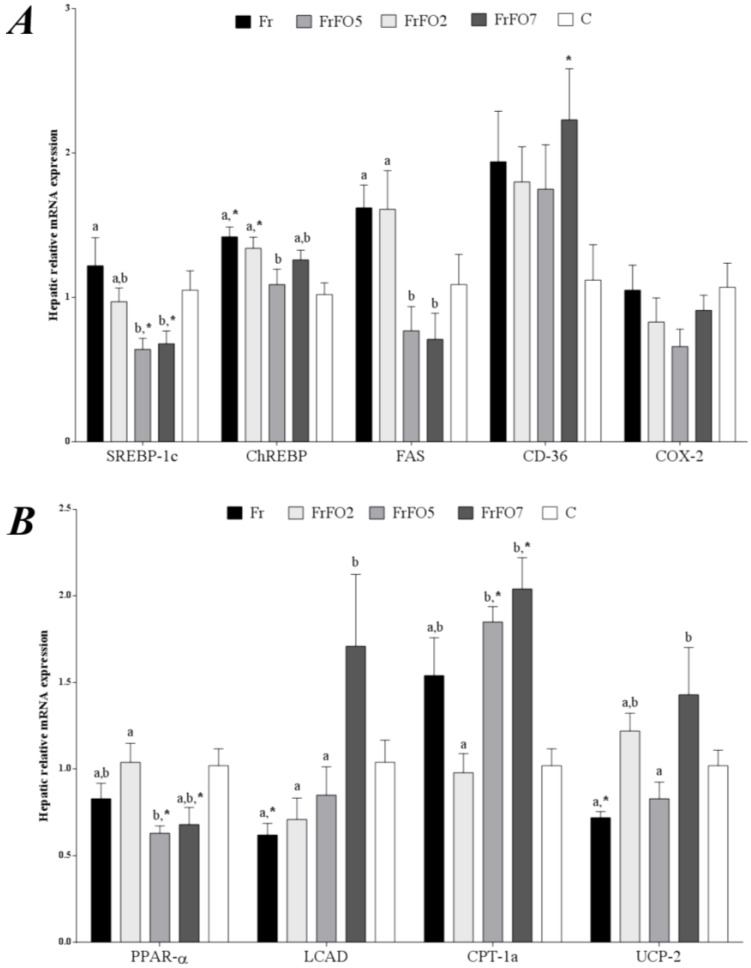

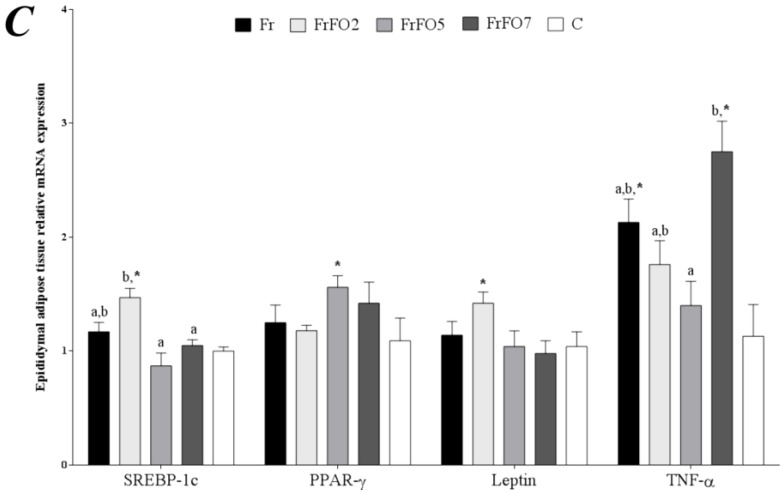
Relative mRNA expression of genes involved in fatty acid metabolism in liver (**A**,**B**) and epididymal adipose tissue (**C**). Data are mean ± SEM. Data for the four groups receiving fructose were compared by ANOVA with Tukey’s test; ^a,b^ values across a row not sharing a superscript letter are significantly different (*p* < 0.05). Data for the control group were compared to each group receiving fructose by unpaired *t* test; * indicates that the fructose group is different from control (*p* < 0.05). SREBP-1c—sterol regulatory element binding protein 1c; ChREBP—carbohydrate regulatory element binding protein; FAS—fatty acid synthase; PPAR-α—peroxisome proliferator activated receptor α; LCAD—long chain acyl CoA dehydrogenase; CPT-1c—carnitine palmitoyl-CoA acyltransferase 1c; CD-36—cluster of differentiation 36; COX-2—cyclooxygenase 2; UCP-2—uncoupling protein 2; PPARγ—peroxisome proliferator activated receptor γ; TNF-α—tumor necrosis factor α. Fr—fructose group; FrFO2—fructose and 2% fish oil group; FrFO5—fructose and 5% fish oil group; FrFO7—fructose and 7% fish oil group; C—control group.

Fr showed higher expression of hepatic lipogenic genes compared with some other groups (Figure 2). For example, mRNA for the transcription factors sterol regulatory element binding protein (SREBP)-1c and carbohydrate regulatory element binding protein (ChREBP), which regulate energy balance, were high in Fr (Figure 2). Fish oil at 5% resulted in lower expression of these transcription factors than seen in Fr. Furthermore, the higher intakes of fish oil resulted in lower fatty acid synthase (FAS) mRNA and higher carnitine palmitoyl-CoA acyltransferase (CPT)-1c mRNA compared to Fr (Figure 2). Additionally, uncoupling protein (UCP)-2 was higher in FrFO7. Peroxisome proliferator activated receptor (PPAR)-α mRNA was lower in FrFO5 and FrFO7 compared to C group for both and to Fr for FrFO5 (Figure 2).

### 2.4. Adipose Tissue Gene Expression

FrFO2 had higher adipose SREBP-1c expression compared to FrFO7 and C (Figure 2). Tumor necrosis factor (TNF)-α expression was higher in FrFO7 compared to FrFO5 and C. Leptin mRNA expression was not correlated with plasma leptin (*r* = 0.05, *p* = 0.76) or total adipose tissue (*r* = 0.23, *p* = 0.17).

### 2.5. Fatty acid Profile of Liver and Adipose Tissue

Fr had higher liver and adipose tissue palmitic acid and oleic acid compared to C (Table 2). Including fish oil in the diet promoted *n*-3 fatty acid incorporation (both EPA and DHA) in both liver and adipose tissue in a dose-dependent fashion (Table 2).

As expected, the liver *n*-6/*n*-3 fatty acid ratio was lower in FrFO5 and FrFO7 compared to the other groups (Table 2). Despite the difference in fish oil consumption, FrFO5 and FrFO7 did not show significant differences in hepatic *n*-3 fatty acid incorporation. However, in adipose tissue *n*-3 fatty acids were higher in FrFO7 than FrFO5.

**Table 2:** Liver and adipose tissue fatty acid composition (wt % of total methyl esters).

Fatty acid	Fr	FrFO2	FrFO5	FrFO7	C
**Liver**					
14:0	0.9 ± 0.1 ^a,^*	0.6 ± 0.1 ^a,b^	0.5 ± 0.04 ^b^	0.7 ± 0.1 ^a,b^	0.5 ± 0.2
16:0	29.1 ± 1.0 ^a^	24.2 ± 1.3 ^b^	23.4 ± 0.6 ^b^	26.4 ± 0.7 ^a,b^	25.7 ± 3.4
18:0	12.9 ± 0.9	15.4 ± 1.0	14.6 ± 0.5	14.6 ± 1.3	14.3 ± 3.5
Total SFA	43.2 ± 0.4 ^a^	40.7 ± 0.8 ^a,b^	39.1 ± 0.4 ^b^	42.6 ± 0.8 ^a^	41.0 ± 3.3
16:1	5.1 ± 0.6 *	3.5 ± 0.6	3.2 ± 0.5	4.6 ± 0.7 *	1.9 ± 1.1
18:1*n*-9	23.0 ± 1.1 ^a,^*	17.0 ± 2.0 ^a,b^	14.8 ± 1.4 ^b^	16.3 ± 1.6 ^b^	16.5 ± 5.1
Total MUFA	28.3 ± 1.8 ^a,^*	20.7 ± 2.5 ^a,b^	18.3 ± 1.9 ^b^	24.6 ± 3.3 ^a,b^	18.5 ± 6.0
18:2*n*-6	11.6 ± 0.7 ^a,^*	14.1 ± 1.0 ^a,b,^*	16.0 ± 1.3 ^b,^*	11.0 ± 0.8 ^a,^*	21.9 ± 3.1
18:3*n*-3	0.4 ± 0.04 ^a,^*	0.5 ± 0.04 ^a,b,^*	0.8 ± 0.1 ^c^	0.7 ± 0.1 ^b,c,^*	0.8 ± 0.1
20:4*n*-6	8.1 ± 0.8 ^a^	13.1 ± 1.4 ^b^	11.1 ± 0.5 ^a,b^	8.6 ± 0.8 ^a^	10.4 ± 5.3
20:5*n*-3	0.1 ± 0.04 ^a^	0.9 ± 0.1 ^a,^*	2.9 ± 0.2 ^b,^*	3.8 ± 0.5 ^b,^*	0.2 ± 0.1
22:6*n*-3	1.6 ± 0.2 ^a^	3.9 ± 0.4 ^b,^*	5.3 ± 0.4 ^b,c,^*	5.4 ± 0.4 ^c,^*	1.3 ± 0.5
Total PUFA	22.1 ± 1.8 ^a,^*	33.1 ± 2.9 ^b^	37.1 ± 2.0 ^b^	30.1 ± 1.7 ^a,b^	35.2 ± 6.8
*n*-6/*n*-3	9.7 ± 0.6 ^a,^*	5.2 ± 0.2 ^b,^*	3.1 ± 0.2 ^c,^*	2.1 ± 0.2 ^c,^*	14.3 ± 3.3
**Epididymal Adipose Tissue**					
14:0	1.20 ± 0.02 ^a^	1.3 ± 0.02 ^a,^*	1.6 ± 0.04 ^b,^*	2.1 ± 0.1 ^c,^*	1.5 ± 0.05
16:0	21.7 ± 0.3	21.3 ± 0.5	21.8 ± 0.6	23.0 ± 0.7	21.2 ± 1.20
18:0	2.1 ± 0.3 ^a^	2.5 ± 0.04 ^a,b^	2.7 ± 0.1 ^b^	2.6 ± 0.1 ^a,b^	2.5 ± 0.23
Total SFA	25.4 ± 0.4 ^a^	25.4 ± 0.5 ^a^	26.7 ± 0.6 ^a,b^	28.1 ± 0.7 ^b,^*	25.1 ± 1.36
16:1	5.0 ± 0.3 ^a^	5.1 ± 0.3 ^a^	5.2 ± 0.3 ^a^	6.7 ± 0.4 ^b^	5.3 ± 1.18
18:1*n*-9	27.7 ± 0.2 ^a,b,^*	28.3 ± 0.6 ^b,^*	26.8 ± 0.2 ^a,c,^*	25.5 ± 0.3 ^c^	25.4 ± 0.91
Total MUFA	32.9 ± 0.2 *	33.6 ± 0.6 *	32.3 ± 0.5	32.7 ± 0.5	31.0 ± 1.41
18:2*n*-6	32.5 ± 0.4 ^a,^*	31.9 ± 0.8 ^a,^*	30.4 ± 0.8 ^a,^*	25.9 ± 1.5 ^b,^*	36.1 ± 1.59
18:3*n*-3	2.8 ± 0.1 ^a^	2.6 ± 0.1 ^a^	2.5 ± 0.1 ^a,^*	2.0 ± 0.1 ^b,^*	3.0 ± 0.19
20:4*n-*6	0.52 ± 0.06	0.4 ± 0.05	0.39 ± 0.01	0.50 ± 0.05	0.42 ± 0.15
20:5*n-*3	0.04 ± 0.01 ^a^	0.13 ± 0.01 ^a,^*	0.39 ± 0.02 ^b,^*	0.98 ± 0.11 ^c,^*	0.04 ± 0.02
22:6*n*-3	0.11 ± 0.02 ^a^	0.28 ± 0.04 ^a,^*	0.88 ± 0.04 ^b,^*	2.26 ± 0.24 ^c,^*	0.06 ± 0.03
Total PUFA	36.3 ± 0.4 ^a,^*	35.7 ± 0.9 ^a,^*	34.82 ± 0.8 ^a,b,^*	31.9 ± 1.3 ^b,^*	39.9 ± 1.65
*n*-6/*n*-3	11.4 ± 0.3 ^a^	10.8 ± 0.4 ^a,^*	8.18 ± 0.2 ^b,^*	5.3 ± 0.5 ^c,^*	12.0 ± 0.76

Data are mean ± SEM. Data for the four groups receiving fructose were compared by ANOVA with Tukey’s test; ^a,b,c^ values across a row not sharing a superscript letter are significantly different (*p* < 0.05). Data for the control group were compared to each group receiving fructose by unpaired *t* test; * Indicates that the fructose group is different from control (*p* < 0.05). SFA—saturated fatty acids; MUFA—monounsaturated fatty acids; PUFA—polyunsaturated fatty acids. Fr—fructose group; FrFO2—fructose and 2% fish oil group; FrFO5—fructose and 5% fish oil group; FrFO7—fructose and 7% fish oil; C—control group.

### 2.6. Liver DHA Is Inversely Correlated to Liver TAG and FAS Expression

**Figure 3:**
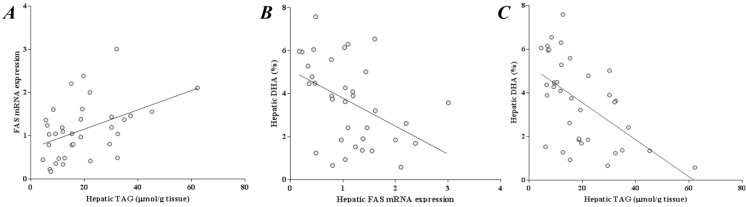
Correlations between (**A**) hepatic triacylglycerol (TAG) and fatty acid synthase (FAS) mRNA expression (*r* = 0.44; *p* < 0.01); (**B**) hepatic docosahexaenoic acid (DHA) (%) and FAS mRNA expression (*r* = −0.44; *p* < 0.01) and (**C**) hepatic DHA (%) and hepatic TAG (*r* = −0.56; *p* < 0.01).

Pearson correlation analysis showed a positive association between liver TAG and FAS expression (Figure 3). Liver DHA was inversely associated to both these parameters (Figure 3).

## 3. Discussion

Fish oil, as a source of the long chain *n*-3 fatty acids EPA and DHA, was able to treat the adverse effects generated by a fructose-rich diet. In the present study, a diet with a typical Western *n*-6/*n*-3 fatty acid ratio (Fr and Control groups, ~10) was compared to one with an ideal ratio (FrFO2 group, ~4) and two with low ratios (FrFO5 and FrFO7 groups, ~1.4 and 0.7, respectively) in a metabolic syndrome model. In this situation of a high amount of simple carbohydrate, the dietary *n*-6/*n*-3 ratio of 1.4 in the FrFO5 group showed the best metabolic response. The FrFO5 group ingested ~330 mg of EPA and DHA per day. This is about 15% of total fat intake in these animals. The American Heart Association recommends 2 g to 4 g of EPA + DHA to lower serum TAG in adult humans [15]. In this situation, in an adult consuming a typical diet with 80 g of fat per day, the *n*-3 fatty acids would provide about 4.5% of fat intake. Thus, the amount of *n*-3 fatty acids provided in the FrFO5 and FrFO7 diets used in the current study is higher than would be seen in most human settings. The amount in the FrFO2 diet was more similar to possible human settings (~7% of fat as EPA + DHA) but this amount was not effective in the current study. 

When compared to FrFO7, FrFO5 group had the advantage of not inducing an increase in hepatic thiobarbituric acid reactive species (TBARS), UCP-2 and TNF-α gene expression. Despite the amount of fish oil in the diet, FrFO5 group presented the same EPA and DHA incorporation in the liver as FrFO7. Thus, the best *n*-6/*n*-3 fatty acid ratio and EPA and DHA amount to reverse the abnormalities caused by a high fructose diet were offered by the 5% fish oil diet.

As expected, a high fructose diet generated an increase in liver TAG, liver weight, serum AST, IR, and hepatic expression of FAS, SREBP-1c and ChREBP. A diet that included fish oil so that the *n*-6/*n*-3 fatty acid ratio was ~1.4 reversed IR and hepatic TAG accumulation without increasing markers of oxidative stress in rats receiving the high fructose diet. These changes were associated with lower expression of mRNA for SREBP-1c and ChREBP, transcription factors involved in lipogenesis, and of FAS mRNA, and with high incorporation of EPA and DHA in liver and adipose tissue. These findings suggest that *n*-3 fatty acids reverse the adverse metabolic effects of a high fructose diet by reversing the fructose-induced upregulation of lipogenic gene expression.

Steatotic livers of animal models and humans present an increase in the *n*-6/*n*-3 ratio and a decrease in DHA content [12,16]. A recent study suggested that treating patients with non-alcoholic fatty liver disease with *n*-3 fatty acids can lower liver fat and demonstrated an inverse relation between erythrocyte DHA enrichment and liver fat loss [17]. In the current study, hepatic DHA was inversely correlated to hepatic TAG and hepatic FAS expression. These observations suggest that the effect of fish oil involves DHA and is through down regulation of *de novo* fatty acid synthesis resulting in lower liver fat.

In the current study, a high fructose diet did not affect blood glucose but induced a high insulin concentration resulting in a higher HOMA-IR index for Fr. HOMA-IR was associated with hepatic TAG, showing the close relation between hepatic fatty liver and insulin resistance. The higher doses of fish oil were able to prevent this adverse effect of fructose. Insulin acts partly through SREBP-1c to regulate lipogenic and glycolytic pathways [18]. Insulin and glucose induce ChREBP expression and EPA and DHA were able to decrease ChREBP, SREBP-1c, FAS and L-pyruvate kinase gene expression in cultures of hepatocytes [19]. In accordance with our observations that fish oil can reverse the adverse metabolic effects of fructose, rats fed a sucrose rich diet and fish oil presented normal insulin action compared to those on a sucrose rich-diet [20]. Fish oil was also able to improve glucose phosphorylation and glucose transporter (GLUT)-4 in skeletal muscle in that model [20].

Rats fed an isocaloric sucrose rich diet for three weeks showed hyperinsulinemia, hypertriglyceridemia, increase in free fatty acids and normal serum glucose. After six months, animals developed severe glucose intolerance with normal insulin levels [21]. Furthermore, fructose can increase FAS expression more than glucose [22]. Also, insulin promotes hepatic FAS expression through SREBP-1c activation [23]. In the present study, fructose induced IR, and increased FAS and SREBP expression.

Compared to sunflower oil, olive oil and coconut oil, fish oil reduced serum TAG and cholesterol and increased secretion of cholesterol, phospholipids and bile acids in rats [24]; however cholesterol was secreted mostly in the form of HDL [24]. The *n*-3 fatty acids appear to stimulate the secretion of cholesterol into bile salts [25], which may explain why there is no increase in serum cholesterol after fish oil provision. 

Serum TAG, total cholesterol and HDL cholesterol were higher in Fr and FrFO2 groups; 5% and 7% fish oil reversed these alterations. Also, these groups presented lower non-HDL cholesterol, which indicates a decrease in more atherogenic lipoproteins [26]. High carbohydrate diets provoke hypertriglyceridemia in mice compared to a high-fat diet [27]. Fructose generates higher serum TAG concentration compared to glucose due to an increase in pyruvate dehydrogenase activity [28]. High doses of fructose also seem to increase total cholesterol and LDL cholesterol in humans, as shown by a recent meta-analysis [29].

Also, fish oil can increase leptin and adiponectin secretion by adipose tissue, which promotes better insulin sensitivity [30]. As expected, serum leptin had a positive association with quantity of adipose tissue. In the current study, fish oil decreased serum leptin levels, but only FrFO2 showed less leptin secretion by adipose tissue, as demonstrated by the leptin/adipose tissue ratio. Furthermore, FrFO2 group presented the highest leptin expression pointing to an endogenous control to upregulate leptin secretion.

Compared to dorsolumbar fat, the effects of *n*-3 fatty acids are more pronounced in the epididymal adipose tissue in which they are able to increase lipid catabolism and decrease adipogenesis [31]. We found a lower percentage of adipose tissue (sum of epididymal and retroperitoneal adipose tissue) with the highest intake of fish oil. Larger quantities of fish oil were effective in reducing adipose tissue weight in mice [32]. However, LDL receptor knockout mice fed a 6% fish oil diet showed increased adipose tissue. These mice had higher cholesterol in adipose tissue, but showed less inflammation, atherosclerosis and hepatic steatosis [33]. Therefore, the decrease in adipose tissue mass seems to be linked to the amount of fish oil in the diet.

FAS may be involved in synthesis of an endogenous PPAR-α ligand, 1-palmitoyl-2-oleoly-sn-glycerol-3-phosphocholine (16:0/18:1-GPC) [21]. FAS knockout mice showed a decrease in 16:0/18:1-GPC and lower liver PPAR-α expression [21]. In the present work, hepatic PPAR-α mRNA was lower in the groups receiving the highest amounts of fish oil. Therefore, the effect of *n*-3 fatty acids on hepatic FAS expression may be linked with the lower hepatic PPAR- α expression.

A decrease in the expression of genes related to production of another endogenous PPAR-α activator was reported in female mice fed a high fish oil diet (60% of total energy intake) for six months. These animals also displayed an increase in fat oxidation, antioxidants and immune reaction genes and a decrease in cholesterol and fatty acid synthesis, transcription factors (such as SREBP-1c) and reactive oxygen species (ROS) [6]. The authors suggested the reduction in cytochrome *P*-450 17-alpha hydroxylase/C17–20 lyase gene expression in fish oil fed mice and the consequent decrease in dehydroepiandrosterone, a PPAR-α activator hormone, was one mechanism responsible for the decreased endogenous PPAR-α activator [6]. These results indicate an adaptive effect to fish oil, decreasing genes related to ROS production and increasing antioxidant and β-oxidative genes.

The FrOP7 group showed lower FRAP, indicating lower serum antioxidant capacity. The groups that received fish oil had higher total serum hydroperoxide concentrations. FrOP7 also showed increased liver TBARS, indicating lipid peroxidation. Because of their higher number of double bonds *n*-3 fatty acids have more susceptibility to lipid peroxidation compared with other fatty acids [34,35]. Similar patterns were found in cultures of rat hepatocytes and, in response to this, animals treated with fish oil had an up regulation of the antioxidant system [6,36,37,38]. Adipose tissue TNF-α was increased in FrFO7. In cultured macrophages *n*-3 fatty acids increased TNF-α and decreased IL-10 secretion [39], while female BalbC mice fed 18% fish oil also presented an increase in TNF-α secretion by splenic macrophages [40]. Human adipocytes also showed increased TNF-α mRNA expression when treated with DHA [41]. These observations seem to be explained by a decrease of a TNF-α suppressor, prostaglandin E_2_, caused by *n*-3 fatty acids [42].

## 4. Experimental Section

### 4.1. Chemicals

Chromatography grade chemicals were used for fatty acid (FA) quantification and other procedures. Standard and biochemical reagents were obtained from Sigma Chemical Co. (St. Louis, MO, USA).

### 4.2. Animals and Diets

Thirty-eight male Wistar rats (weighing ~200 g) were obtained from the Central Animal Care of Ribeirão Preto Campus, University of São Paulo. The animals were kept on a 12 h light–12 h dark cycle at an average temperature of 22 °C. Food (AIN-93G [43]) and water were provided *ad libitum*. All procedures followed the guidelines of the Brazilian College of Experiments with Animals and were approved by the Institution’s Ethics Committee of Animal Experimentation on 29 March 2010 (protocol number 013/2010). Initially, animals were randomly divided into 2 experimental groups: fructose group (Fr; *n* = 32) and control group (C, *n* = 6). C received the AIN-93 diet [43] containing 20% protein (casein), 63% carbohydrate (53% starch and 10% sucrose), 7% fat (soybean oil), 5% fiber, 3.5% AIN-93G mineral mix, 1% AIN-93G vitamin mix, 0.3% L-cystine, 0.25% choline, and 0.002% di-tert-butyl methyl phenol (BHT). Fr received the AIN-93 diet with 63% fructose instead of starch and sucrose for 60 days. After this, 24 rats from Fr were randomly assigned to 3 dietary groups of 8 rats each: fructose and 2% fish oil (FrFO2), which received 2% fish oil and 5% soybean oil; fructose and 5% fish oil (FrFO5), fed with 5% fish oil and 2% soybean oil; and fructose and 7% fish oil (FrFO7), provided with 7% fish oil. The remaining diet components followed the AIN-93 recommendations. The diets were isocaloric and the animals received fresh food every 2 days. Animal weight and food intake were recorded weekly throughout the study.

Fish oil was purchased from Campestre Industry and Trade of Vegetal Oils^®^ (São Bernardo do Campo, Brazil). Vitamin, mineral mix, choline and L-cystine were purchased from Rhoster (Rhoster, Araçoiaba da Serra, Brazil). The fatty acid composition of the diets is given in Table 3.

**Table 3:** Fatty acid composition of the diets used.

Fatty Acid	C and Fr	FrFO2	FrFO5	FrFO7
14:0	0.1	1.86	4.5	6.3
16:0	10.6	14.63	19.9	23.6
16:1	ND	1.92	4.8	6.7
17:0	0.1	0.33	0.7	0.9
17:1	0.1	0.29	0.6	0.9
18:0	2.9	3.64	4.6	5.3
18:1*n*-9	27.1	23.53	16.4	12.1
18:2*n*-6	52.0	42.65	25.1	14.3
18:3*n*-3	5.2	4.50	3.1	2.2
20:1*n*-9	0.3	0.21	0.1	ND
20:2	0.2	0.35	0.5	0.6
20:3*n*-6	ND	0.06	0.1	0.2
21:0	ND	0.05	0.1	0.2
20:3*n*-3	ND	0.02	0.01	0.1
20:4*n*-6	ND	0.41	1.0	1.4
20:5*n*-3	0.4	3.04	7.0	9.6
22:1*n*-9	ND	0.10	0.2	0.3
24:0	0.1	0.42	0.9	1.2
22:6*n*-3	ND	3.62	9.0	12.6
Other	0.9	4.27	9.3	12.6
Total *n*-6	52.0	43.11	26.2	15.9
Total *n*-3	5.6	11.17	19.1	24.5
*n*-6/*n*-3	9.25	3.86	1.37	0.65

Values are means as weight percentage (w/w) of the total fatty acid methyl esters. ND, not detected. C—control group; Fr—fructose group; FrFO2—fructose and 2% fish oil group; FrFO5—fructose and 5% fish oil group; FrFO7—fructose and 7% fish oil group.

At the end of 90 days the animals were fasted for 10 h and then sacrificed by decapitation. Blood was collected and centrifuged at 3000 rpm and 4 °C. Serum was stored at −80 °C until analysis. Livers, retroperitoneal and epididymal adipose tissues were removed, weighed and freeze-clamped with aluminum tongs. Small pieces of liver and epididymal adipose tissue were kept in a RNA stabilizer solution (RNAlater^®^, Qiagen, Valencia, CA, USA) until RNA extraction. To avoid exogenous RNase contamination, all materials used during the sacrifice were treated with Rnase away™ decontamination reagent (Invitrogen, Life Technologies, Carlsbad, CA, USA).

### 4.3. Biochemical Analyses

Leptin and insulin were measured by ELISA kits for rats (RayBio^®^ Rat Leptin ELISA and Rat Insulin ELISA Kit, Shibayagi Co. Ltd., Shibukawa, Japan). Triacylglycerols (TAG), total cholesterol, high-density lipoprotein (HDL) cholesterol, glucose, and aspartate aminotransferase (AST) were determined using commercial kits (Labtest Diagnóstica S.A., Vista Alegre, Brazil). Non-HDL cholesterol was calculated as: total cholesterol minus HDL cholesterol. The homeostatic model assessment (HOMA1-IR) index was calculated following the formula proposed by Matthews *et al.* [44], which uses fasting glucose (mmol/L) multiplied by fasting insulin (μUI/L) divided by 22.5. Liver TAG and total cholesterol were measured by commercial kits after hepatic lipid extraction [45]. Serum and liver protein, used to normalize oxidative stress parameters, were measured according to Lowry *et al.* [46].

Liver fatty acids were extracted and derivatized to methyl esters by a direct transesterification method adapted from Lewis *et al.* [47] and methyl esters separated on a gas chromatograph (Shimadzu Europe, Duisburg, Germany) equipped with an AOC-20i auto-injector (Shimadzu Europe, Duisburg, Germany) using a fused silica SP-2560 column (100 m, 0.25 mm I.D., film thickness 0.20 μm). Helium was used as carrier gas and make-up gas was air. Synthetic air was used for flame ionization detection at 250 °C. Injections were made in the split mode. Fatty acid methyl ester retention times were determined by comparison with external standards (Supelco 37 component FAME Mix; Supelco, Bellefonte, PA, USA).

The ferric reducing antioxidant power (FRAP) assay was used to measure serum antioxidant capacity [48]. The FRAP assay solution was made by mixing an acetate buffer (300 mM), ferric 2,4,6-s-triazine-tripiridil in HCl (40 mM) and aqueous FeCl_3_ (20 mM). Serum (10 μL) was incubated with FRAP assay solution at 37 °C for 4 min and then the absorbance was measured at 593 nm. Serum total hydroperoxides were analyzed in accordance with Galli *et al.* [49], in which the oxidation of ferrous ion to ferric ion by serum peroxides is measured. For this, a solution of xylenol orange in methanol and ferrous sulfate in 10 mM sulfuric acid was prepared and added to 100 μL serum and kept in the dark for 30 min. Then the absorbance was read at 560 nm. Serum and liver lipid peroxidation was measured by the determination of thiobarbituric acid reactive species (TBARS) according to the method of Mihara and Uchiyama [50]. Reduced glutathione (GSH) in liver and serum was determined by the method of Sedlack and Lindsay [51]. Liver glutathione peroxidase and glutathione reductase activities were measured by the method reported by Paglia *et al.* [52].

### 4.4. mRNA Extraction and Quantification

Liver mRNA was extracted using TRIzol (Trizol reagent—Invitrogen, Life Technologies, Carlsbad, CA, USA) following the manufacturer’s instructions. A commercial kit was used to extract mRNA from epididymal adipose tissue (Illustra RNAspin Mini RNA isolation, GE Healthcare Life Sciences, Uppsala, Sweden). cDNA was synthetized with a commercial kit containing random primers (High Capacity cDNA Reverse Transcription Kit, Life Technologies). Sybr Green master mix (Power SYBR^®^ Green PCR Master Mix, Applied Biosystems, Carlsbad, CA, USA) was used to perform real-time qPCR using an ABI Prism 7500 Sequence Detection System instrument (Applied Biosystems, Carlsbad, CA, USA) and specific primers for each gene (Invitrogen, Life Technologies, Carlsbad, CA, USA). Relative RNA expression was standardized to the endogenous housekeeping gene 18S and calculated using the ΔΔC_T_ method. No differences among groups were observed for the 18S expression. The sequence of the sense and antisense primers used for amplification were:

SREBP-1c—*F* AGCACAGCAACCAGAAACTC, *R* AGGTTTCATGCCCTCCATAG
ChREBP—*F* CTTCAAAGGCCTCAAGTTGC; *R* TTCCTCCGTTGCACATACTG
FAS—*F* TCTGATCAGTGGCCTCCTTAAC, *R* CAGTGCTGAGATGTGGGAATAC
PPAR-α—*F* GCAATGCACTGAACATCGAG, *R* TCTTGCAGCTTCGATCACAC
LCAD—*F* AAACAGTCGCACACATCCAG, *R* CCAGACGTTTGGTTTCATGC
CPT-1α—*F* TTGACTCTTTCGGCAAAGGC, *R* TCCTTGTAATGTGCGAGCTG
CD-36—*F* TGGATGTGGAACCCATAACTGG, *R* TCCCAGTCTCATTTAGCCACAG
COX-2—*F* CCAGTATCAGAACCGCATTG, *R* TGAGCAAGTCCGTGTTCAAG
UCP-2—*F* ACAAGACCATTGCACGAGAG, *R* TGGCATTTCGGGCAACATTG
Leptin—*F* CAAGCTGTGCCTATCCACAAAG, *R* ATGAAGTCCAAACCGGTGAC
TNF-α—*F* TGCCTCAGCCTCTTCTCATTC, *R* TGGGAACTTCTCCTCCTTGTTG
PPAR-γ—*F* TGCTTGTGAAGGATGCAAGG, *R* GCACTTCTGAAACCGACAGTAC
18S—*F* GATAAGCCCAAGCTCAATCG, *R* TTCTGGAGTAGCGGACATTG

### 4.5. Statistical Analysis

Data are expressed as mean ± standard error of the mean (SEM). One-way analysis of variance (ANOVA) and Tukey post-test were used for data analysis using the GraphPad Prism version 5.00 for Windows (GraphPad Software, San Diego, CA, USA) to compare groups that received fructose as the only carbohydrate source. Unpaired *t*-test was used for comparisons between control and experimental (*i.e.*, those receiving fructose) groups. Pearson correlation test were used to determine association between variables. Differences were considered significant when *p* < 0.05.

## 5. Conclusions

In summary, including fish oil in the diet was able to reverse a number of the adverse metabolic effects of a high fructose diet and these beneficial effects involved an alteration in gene expression profiles. Therefore, fish oil may be useful to treat metabolic syndrome comorbidities. A balanced *n*-6/*n*-3 ratio, with the *n*-3 fatty acids provided as EPA and DHA, alters metabolic pathways and modulates gene expression resulting in better insulin sensitivity, lower dyslipidaemia and hepatic TAG without increasing oxidative stress. These findings provide a rational basis for the use of EPA and DHA as an alternative treatment for patients with metabolic syndrome.
